# Supplementary material for: Impaired Well-Being and Insomnia as Residuals of Resolved Medical Conditions: Survey in the Italian Population
Source: Int J Environ Res Public Health. 2024 Jan 24;21(2):129. doi: 10.3390/ijerph21020129 (PMC10888320; doi:10.3390/ijerph21020129)
Supplement: Supplementary file 1 [file ijerph-21-00129-s001.zip › ijerph-2688576-supplementary_rev.pdf]

Supplementary Materials files

Supplementary tables

Table S1. Contingency table of the association between WHO-5 and gender. Pearson's $X^2 = 44.710$; p = 0.

		Males	Females	Total
**WHO-5 > 50**	**Number (%)**	1642 (61.2%)	1503 (52.3%)	3145 (56.6%)
**WHO-5 ≤ 50**	**Number (%)**	1043 (38.8%)	1373 (47.7%)	2416 (43.4%)
**Total**	**Number (%)**	2685	2876	5561

WHO-5, World Health Organization well-being scale-5.

Table S2. Contingency table of the association between ISI and gender. Pearson's $X^2 = 32.941$; p = 0.

		Males	Females	Total
**ISI < 10**	**Number (%)**	2168 (80.7%)	2137 (74.3%)	4305 (77.4%)
**ISI ≥ 10**	**Number (%)**	517 (19.3%)	739 (25.7%)	1256 (22.6%)
**Total**	**Number (%)**	2685	2876	5561

ISI, Insomnia Severity Index.

Table S3. Contingency table of the association between ISI and WHO-5. Pearson's $X^2 = 540.45^{a}$; p = 0.

		ISI < 10	ISI ≥ 10	Total
**WHO-5 > 50**	**Number (%) of subjects within ISI**	2794 (64.9%)	351 (27.9%)	3145 (56.6%)
**WHO-5 ≤ 50**	**Number (%) of subjects within ISI**	1511 (35.1%)	905 (72.1%)	2416 (43.4%)
**Total**	**Number (%) of subjects within ISI**	4305	1256	5561

ISI, Insomnia Severity Index; WHO-5, World Health Organization well-being scale-5.

Table S4. Contingency table of association between ISI categories and WHO-5. Pearson's $X^2 = 698.81$; p = 0.

		ISI 0-7 (no insomnia)	ISI 8-14 (subclinical insomnia)	ISI ≥ 15 (moderate/severe insomnia)	Total
**WHO-5 > 50**	**Number (%) of subjects within ISI categories**	2521 (68.7%)	576 (36.8%)	48 (14.8%)	3145 (56.6%)
**WHO-5 ≤ 50**	**Number (%) of subjects within ISI**	1150 (31.3%)	989 (63.2%)	277 (85.2%)	2416 (43.4%)
**Total**	**Number (%) of subjects within ISI**	3671	1565	325	5561

ISI, Insomnia Severity Index; WHO-5, World Health Organization well-being scale-5.

Table S5. Binary logistic regression model of association between WHO-5 and the number of pathologies.

Variables	Regression weights							
	B	S.E.	Wald	d.f.	O.R.	95% C.I. for O.R.		P value
						Lower bound	Upper bound	
**Age**	-0.01	0.00	2.75	1	0.99	0.98	1	0.09
**Gender**	0.14	0.15	0.98	1	1.16	0.86	1.55	0.32
**Age x gender**	0.00	0.00	0.87	1	1	0.99	1.00	0.35
**Onlypast**			42.32	3				
**Onlypast (n=1)**	0.26	0.06	21.38	1	1.3	1.16	1.46	0.00
**Onlypast (n=2)**	0.31	0.08	13.46	1	1.36	1.15	1.61	0.00
**Onlypast (n=3)**	0.57	0.12	20.89	1	1.78	1.39	2.28	0.00
**Onlypresent**			192.09	3				
**Onlypresent (n=1)**	0.37	0.06	37.67	1	1.44	1.28	1.63	0.00
**Onlypresent (n=2)**	0.65	0.08	65.75	1	1.91	1.63	2.23	0.00
**Onlypresent (n=3)**	1.24	0.09	165.77	1	3.45	2.86	4.17	0.00
**Constant**	-0.78	0.24	10.6	1	0.45			0.00

B, beta coefficient; C.I., confidence intervals; d.f., degrees of freedom; O.R., odds ratio; S.E., standard error.

Table S6. Binary logistic regression model of association between ISI and the number of pathologies.

Variables	Regression weights							
	B	S.E.	Wald	d.f.	O.R.	95% C.I. for O.R.		P value
						Lower bound	Upper bound	
**Age**	-0.02	0	8.93	1	0.98	0.96	0.99	0.00
**Gender**	-0.18	0.22	0.68	1	0.83	0.54	1.28	0.41
**Age x gender**	0.01	0	6.39	1	1.01	1	1.02	0.01
**Only present**			122.3	3				0.00
**Only present (n=1)**	0.41	0.09	21.93	1	1.51	1.27	1.79	0.00
**Only present (n=2)**	0.77	0.11	49.05	1	2.2	1.74	2.69	0.00
**Only present (n=3)**	1.28	0.12	108.7	1	3.61	2.84	4.6	0.00
**Only past**			29.36	3				0.00
**Only past (n=1)**	0.32	0.08	15.01	1	1.37	1.17	1.61	0.00
**Only past (n=2)**	0.46	0.12	15.18	1	1.59	1.26	2.01	0.00
**Only past (n=3)**	0.55	0.17	10.01	1	1.73	1.23	2.43	0.00
**Constant**	-1.26	0.36	12.21	1	0.28			0.00

B, beta coefficient; C.I., confidence intervals; d.f., degrees of freedom; O.R., odds ratio; S.E., standard error.

Table S7. Multinomial logistic regression model of association between ISI categories and the presence of at least one current or resolved pathology.

ISI categories	Variables (d.f.)	Regression weights			
		O.R.*	95% C.I. for O.R.		P value
			Lower Bound	Upper Bound	
**ISI 8-14**	**Age (1)**	0.98	0.97	1.00	0.06
	**Gender (1)**	0.98	0.66	1.46	0.94
	**Age x gender (1)**	1.00	0.99	1.01	0.15
	**Only present (1)**	1.56	1.36	1.79	0.00
	**Only Past (1)**	1.45	1.27	1.66	0.00
**ISI ≥ 15**	**Age (1)**	0.97	0.95	1.00	0.08
	**Gender (1)**	0.85	0.38	1.93	0.7
	**Age x gender (1)**	1.01	0.99	1.03	0.07
	**Only present (1)**	3.08	2.32	4.08	0.00
	**Only past (1)**	1.82	1.42	2.34	0.00

*Reference category is 0-7. C.I., confidence intervals; d.f., degrees of freedom; ISI, Insomnia Severity Index; O.R., odds ratio.

Table S8. Multinomial logistic regression model of association between ISI categories and the number of pathologies.

ISI categories		Regression weights							
		B	S.E.	Wald	d.f.	O.R.	95% C.I. for O.R.		P value
							Lower Bound	Upper Bound	
**ISI 8-14**	**Intercept**	-0.93	0.33	8.07	1				0.00
	**Age**	-0.01	0.007	5.31	1	0.99	0.97	0.99	0.02
	**Gender**	-0.02	0.2	0.01	1	0.98	0.66	1.45	0.90
	**Age x gender**	0.006	0.004	2.04	1	1.00	0.99	1.01	0.15
	**Only past (n=0)**	0[b]			0				
	**Only past (n=1)**	0.52	0.11	21.15	1	1.69	1.35	2.11	0.00
	**Only past (n=2)**	0.34	0.08	19.25	1	1.40	1.20	1.63	0.00
	**Only past (n=3)**	0.36	0.17	4.41	1	1.44	1.02	2.03	0.03
	**Only present (n=0)**	0[b]			0				
	**Only present (n=1)**	0.59	0.11	30.34	1	1.80	1.46	2.22	0.00
	**Only present (n=2)**	0.28	0.08	11.89	1	1.32	1.13	1.55	0.00
	**Only present (n=3)**	0.93	0.12	54.48	1	2.53	1.98	3.24	0.00
**ISI ≥ 15**	**Intercept**	-2.6	0.69	14.25	1				0.00

**Age**	-0.03	0.01	5.77	1	0.97	0.94	0.99	0.01
**Gender**	-0.19	0.4	0.21	1	0.83	0.37	1.84	0.64
**Age x gender**	0.01	0.008	3.15	1	1.01	0.99	1.03	0.07
**Only past (n=0)**	0[b]			0				
**Only past (n=1)**	0.73	0.20	12.42	1	2.08	1.38	3.13	0.00
**Only past (n=2)**	0.56	0.14	14.83	1	1.75	1.31	2.33	0.00
**Only past (n=3)**	0.91	0.28	10.53	1	2.48	1.43	4.30	0.00
**Only present (n=0)**								
**Only present (n=1)**	1.31	0.20	44.39	1	3.7	2.52	5.45	0.00
**Only present (n=2)**	0.74	0.17	19.07	1	2.09	1.50	2.91	0.00
**Only present (n=3)**	2.16	0.20	113.8	1	8.70	5.84	12.94	0.00

B, beta coefficient; C.I., confidence intervals; d.f., degrees of freedom; ISI, Insomnia Severity Index; O.R., odds ratio; S.E., standard error.

Supplementary Figures

IPSAD® 2017
Italian Population Survey on Alcohol and Other Drugs

General Health Section

D14. Has your doctor ever told you that you suffer from one or more of the following diseases/health conditions?
(Mark all appropriate options, even morethan one per row)

	No	I suffered in the past	I suffer now
a) Diabetes	o	o	o
b) High blood pressure	o	o	o
c) Myocardial infarction, angina or any ischemic cardiovascular diseases	o	o	o
d) Stroke or other cerebrovascular diseases	o	o	o
e) Other heart diseases (arrhythmias, heart valve diseases, heart infection, congenital diseases)	o	o	o
f) High cholesterol	o	o	o
g) Thyroid disorders	o	o	o
h) Tumor (including lymphoma or leukemia)	o	o	o
i) Psychiatric disorders (including anxiety, depression)	o	o	o
l) Joint and rheumatic pain (including fibromyalgia, rheumatoid arthritis etc.)	o	o	o
m) Diseases of the skin (psoriasis, eczema, etc.)	o	o	o
n) Sleep apnea	o	o	o
o) Cognitive Impairment (memory loss, Parkinson's disease, senile dementia, etc.)	o	o	o

Figure S1. General Health section of the IPSAD® 2017 questionnaire, Item D 14.

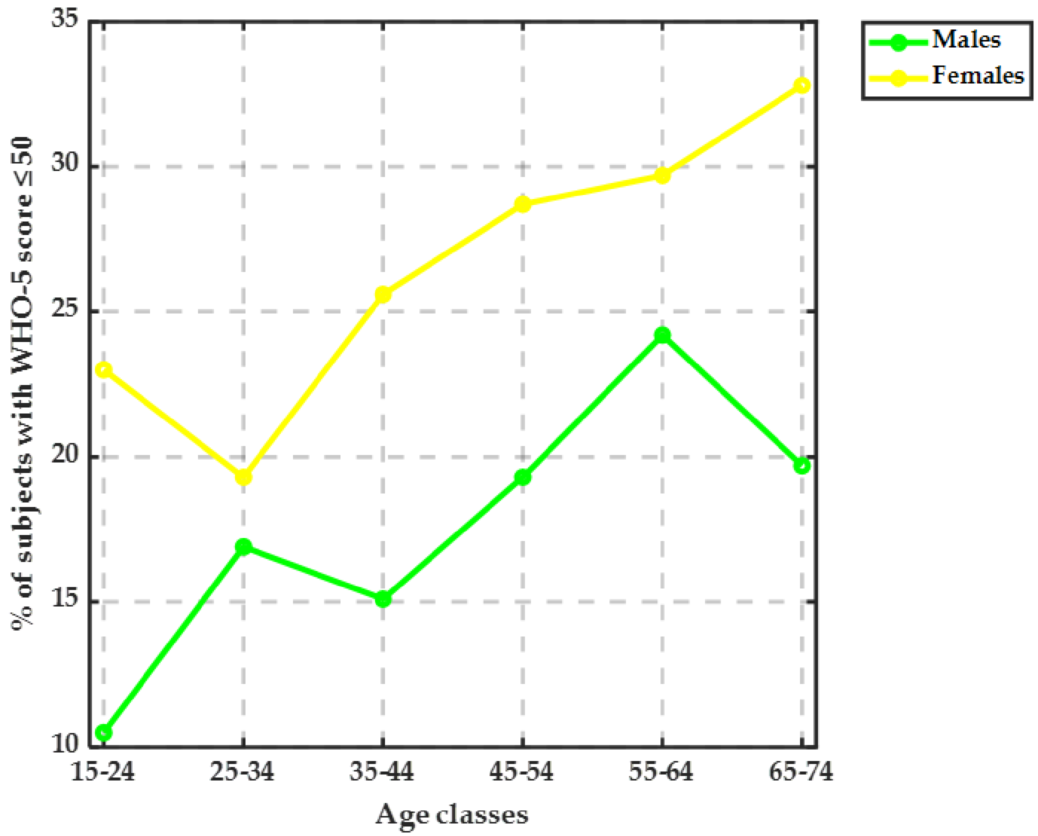

Figure S2. Effect of age by gender interaction on well-being level.

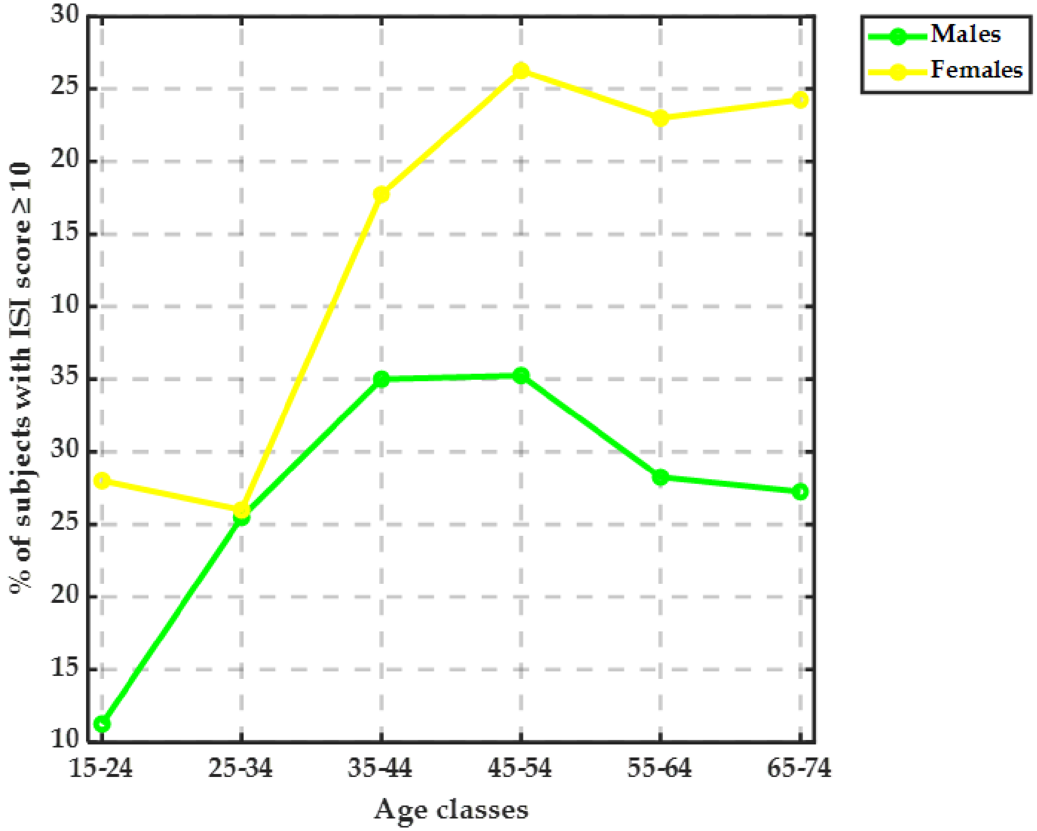

Figure S3. Effect of age by gender interaction on insomnia level.
